# A Community-Based Music Therapy Support Group for People With Alzheimer's Disease and Their Caregivers: A Sustainable Partnership Model

**DOI:** 10.3389/fmed.2018.00293

**Published:** 2018-11-06

**Authors:** Robin Rio

**Affiliations:** Arizona State University, Tempe, AZ, United States

**Keywords:** music therapy, Alzheimer's, dementia, caregiver, support, group

## Abstract

People with Alzheimer's and related dementias and their family caregivers who reside at home have unique strengths and needs. They have the strengths of being in a place with which they are familiar, with people whom they are in close relationship. Often it is the spouse who provides the primary care of their loved one, and as the disease progresses both members of the couple are at risk for depression, isolation, and decreased contact with peers and community networks that serve to help maintain sociocultural, intellectual, physical, sensory, and spiritual needs. The person with Alzheimer's Disease (AD) loses life skills and sense of self as their memory loss worsens, and the caregiver-loved one, whether they are the spouse, relative, or close companion, becomes increasingly burdened physically and emotionally. Meaningful support through a community-based peer group helps meet the needs of the person with dementia and their caregiver from the first symptoms to the later stages of AD, through a carefully designed music therapy program tailored to preferences, culture, and ability. The music therapist working in the community provides practical leadership in coordination with local agencies, understands the needs of the person with dementia and their caregiver from a cultural and psychosocial perspective, and is creatively equipped in all facets of musical engagement for health and wellness, and fosters cognitive/intellectual, socio-emotional, physical, and spiritual support. The MT support group was found to relieve some of the strain on caregivers by allowing for greater emotional support through relationships with peers and professionals, and through the increase of meaningful interactions with their loved one with dementia. Through enjoyment of shared, pleasurable music experiences that stimulate memories, movement, language, and socialization, the person with AD and their caregiver developed a deeper connection with each other, and gained support, creative expression, and comfort from their peer group, as well as practical networking and sharing of resources and information related to their specific health and wellness needs. The community-based MT support group has been replicated twice within the region, and is a promising model for other communities. Formal research is recommended to provide further evidence of the effectiveness of the approach, and to allow for greater accessibility for marginalized people to participate in a program such as this in their own community.

## Introduction

Communities face challenges in meeting the needs of home dwelling seniors experiencing cognitive losses who are aging in place. Social needs become magnified for individuals with Alzheimer's and related dementias (PwD) and their loved ones, and opportunities to engage with others who have similar needs and interests become limited. People with dementia experience difficulty with many aspects of daily living, and these needs impact their care partners. Typically, loss of memory, difficulty with language and communication, and decrease in judgment are issues that affect home life, creating behavioral and psychological symptoms of dementia (BPSD), which are accompanied by physical decline. The care partner needs to find ways to help their loved one manage daily living in the face of these symptoms and behaviors, as they themselves must cope with mounting losses as the disease progresses (1, 2). Caregivers are most often spouses and life partners, but can also be an adult child, a relative or friend, or a professional provider. When living within a strong network of support, combinations of relatives, professionals and close friends rely on one another to provide supervision and care for the PwD. The primary care partner is at a risk for depression and decreased quality of life, and since the majority of people with Alzheimer's and related dementias are already in their later years, the caregivers are likely to be experiencing their own health issues. Taking care of a loved one is perceived as both rewarding and difficult, and causes strain on physical and emotional health. Caregivers are at risk for depression and overuse of alcohol, tobacco and drugs, being overweight, and under stress and worry without adequate resources (3).

For the PwD, research indicates that music therapy has been found to reduce agitation (4–6), reduce Behavioral and Psychological Symptoms of Dementia (BPSD) (7, 8) and decrease anxiety and depression (9). Research shows that participants who engaged in MT improved mood (10) by singing and listening, and through reminiscence MT (11) and listening to 20-min sequences of computer programmed music (9). They improved in their BPSD when involved in an improvisation-based group which took place over 30 sessions across 16 weeks (12), as well as when engaged in singing songs with guitar accompaniment three times per week for 30 min, across 6 weeks (13). A group of French experts examined over 200 articles on MT and dementia, and came to the conclusion that MT can contribute to emotional and social skills, as well as decrease the severity of problematic behaviors while also supporting cognition (14). Professional caregivers have used singing to improve care situations by reducing resistance, such as pulling away, in morning care (15) and in communicating more effectively with the person with advanced dementia (16, 17).

For the home-based caregiver, there is a clear need for improvement to and availability of services, as recently noted in the Alzheimer's Caregiver Support Act [2017–2018; (18)]. Various disciplines recognize how they may support the caregiver of the PwD. Halpin et al. (19) formulated a theory about the socio-emotional adaptation of the PwD. They found that when the caregiver, along with professionals assisting in treatment of the PwD are accurately understanding behaviors and discovering the underlying unmet needs, the PwD can experience an improved mood by adapting to the specific situation. For example, an individual who has previously enjoyed reading and is no longer able to read, the use of an audio book may help to relieve the problem. The socio-emotional adaptation theory can be applied to the community-based support group. If the PwD has enjoyed being a part of a social circle through conversation, music and/or dancing prior to the onset of dementia, but is no longer able to participate in the same way they once were, adapting communication to the mode of singing, playing rhythms or moving to music can help alleviate some frustration caused by deficits in speech and physical mobility, and therefore ease the tension on the caregiver. Ridder et al. (6) examined music therapy components for promoting interactions while Clair (20) found increased mutual engagement between the PwD and their caregiver, and Klein and Silverman (21) found that a songwriting intervention helped with coping skills for the care partner of the PwD by reducing stress, having fun, and improving group cohesiveness and camaraderie while providing psychoeducational counseling. Hanser (22) addressed the needs of the PwD and their family caregiver and described a protocol that was facilitated by the music therapist, improving the psychological health of the PwD and reducing the stress of the caregiver in their home while improving the quality of life, reporting enjoyment in the interactions with loved ones that occurred while reminiscing and making music.

Based on the research and practical experience, the needs of the Person with Dementia and their caregiver are identified.

### Needs of the person with dementia (PwD) their care partner (CP) and (both)

- Assistance in Organizing their Day/Schedule (PwD)
- Assistance with Personal Care, Medication Management, ADL's (PwD)
- Social Support (Both)
- Mood Elevation/Stabilization (Both)
- Opportunities for Reminiscing and Meaningful Conversation and Communication (Both)
- Ability, Awareness and Opportunities to make Choices (PwD)
- Motivation to Participate in Physical Exercise (Both)
- Explanation and Understanding when Confused (PwD)
- Redirection when Wandering or Becoming Agitated or Anxious (PwD)
- Intellectual and Sensory Stimulation (Both)
- Networking Opportunities with other Care Partners (CP)
- Artistic and Creative Outlet (Both)
- Respite from Responsibilities (CP)
- Opportunities to Connect with their Loved One (Both)
- Support of New Roles in Relationships and Changing Identity (Both)
- Access to Experienced Professionals in Dementia Care (Both)
- Coping Strategies (Both)
- Decrease in Stress and Increase in Overall Wellbeing (Both)

The community-based MT support group engages both the PwD and their care partner in a welcoming setting, providing socio-emotional support, engagement in a pleasurable event, opportunities for reminiscing, and active participation in music making. Through singing, instrument playing, movement, and creation of new musical arrangements and improvisations based on in-the-moment needs, feelings and concerns, the PwD is able to participate more easily than traditional, non-adapted conversation and dance. Music experiences are facilitated by professional and student music therapists in the context of a support group where volunteers, family members, and the PwD are involved in the planning and implementation of the sessions. The principles from community music therapy (CoMT) provide an understanding that treatment and health exist within a system, and that the community at risk is not an empowered group (23). As with other CoMT groups, the PwD and their caregiver struggle with having a voice (24), in this case due to: ageism, cognitive losses, decrease in access to services, and a lessening of stature in a social culture that values productivity and youth. The community-based MT support group sessions put focus on the needs of both the Pwd and their caregiver, fostering a team approach. Many of the need areas listed above as need areas for both the PwD and their caregiver become attainable goals through the sessions.

In a supportive group environment, the PwD and their care partner are provided a safe space within a peer group framework to redefine their changing social identity. In addition to health needs and cognition, concerns regarding social policy can be discussed among group members and professionals, taking into account the particular needs of the marginalized group. Initiatives such as the *dementia friendly community* (2018), which include availability and accessibility to healthful programs and services, are discussed within the group, so that the service recipients may have influence on what is offered, and how it is offered. Within the CoMT paradigm is recognition of the human need for connection with others. The role of the music therapist involves facilitating and supporting connection (25) among all members of the community, and with an egalitarian attitude. With this in mind, the caregiver is often referred to as the care partner, recognizing the partnership with the person affected by dementia.

In *Connecting Through Music with People with Dementia: A Guide for Caregivers* (26) the concept of strengthening the musical connection was a central element identified in working with people who needed to find new ways to interact and engage when faced with the challenges of a neurodegenerative disorder, and the individual(s) who most wanted to connect with the PwD was their loved one: their caregiver(s). The need for making musical connections with others becomes even more pronounced when verbal communication and cognitive processes are limited. By literally and figuratively amplifying the expressions of the participants, their voices, gestures, personalities, and memories- each member of the group has an integral role in the shaping of the group music experience. By meeting regularly in a safe space, free of judgment or stigma associated with decrease in ability or difficulty in managing behaviors, the group members are able to support one another through sharing of lived experience, expressing emotions, and using physical gesture and creative processes to communicate with their loved ones as words and memory are failing.

### Developing a community-based MT support group

When looking to develop a creative community support group, an experienced nurse in charge of community engagement at an Alzheimer's research and treatment center approached the author, a university MT program clinic director, to develop a MT group for PwD living in their own home in the community. Specifically, this MT group was initiated in response to a need identified by a member of the Alzheimer's community who was a musician with younger-onset AD, at the request and advocacy of her caregiver-spouse.

To begin this program partnership, each of the stakeholders, including the Alzheimer's research and treatment center, the university and its music therapy program, and the PwD and care partners, first identified unmet needs. These needs contributed to the participants' desire and motivation to invest in the MT program. The goals and mission of the new program aligned with each organization's goals and mission statements. The MT working in a university setting is in an ideal position to nurture a creative community partnership. Moving comfortably between health care and the arts, the MT is able to understand the needs and processes of health agencies and local government entities, as well assess a broad scope of citizens' concerns related to health and access to services. The following represents the goals of the various partners and stakeholders in the MT group, and includes principles from Dementia Friendly America (27).

### Goals of community partners and stakeholders

- Dementia-friendly *healthcare* connects individuals to community services
- The *university* places high value on community engagement and social embeddedness
- The *university MT program* requires supervised clinical placement sites for student learning and community service
- Dementia friendly *government* works to create a culture that helps PwD and their caregivers to thrive and age in place
- Community *residents with dementia and their care partners* desire active participation in health promotion, and safe accessible spaces to interact and create
- A *cooperative model* invests in professionals, volunteers, students, and the *partners share leadership* in providing services and developing the program culture

### Program development

The development of the MT group was client-driven: a highly educated and energetic retired minister sought out community leaders in healthcare, education, and government to take action in providing accessible MT programming for his wife, a retired church musician, who was living in their family home with younger-onset AD. These founding members were proactive in advertising the program to members of an already existing discussion-based support group for caregivers at the Alzheimer's treatment and research center. Having the community member seek the program and provide input into needs of the participants and the meaning it holds for them is a key element of a Community Music Therapy (CoMT) approach. Promoting the program through already established, ongoing events and programs provided effective outreach. The nurse in charge of community engagement at the Alzheimer's center for treatment and research was the referral source, and was present to assist in monthly MT sessions.

Criteria for Participation

- Reside at home
- Are in early to moderate stage of Alzheimer's Disease or related dementia
- Care partner must attend together with the PwD
- Able to tolerate a group setting

### Implementation

The first sessions were held in spring, 2011, at the university's community services building in the largest room of the MT clinic. A piano, guitar, and various percussion instruments from the university MT program were available. At the first monthly meeting, only the initial couple attended. The PwD played piano with the music therapist, and through these interactions a brief and informal music therapy assessment revealed the musical interests of “Jackie,” who became the group's accompanist (28) which allowed her to maintain her identity as a musical leader. As the sessions grew in number, averaging 10-20 participants including the facilitators and volunteers, the format was standardized to allow for participants and volunteers to have more autonomy in leadership within a flexible structure. The session outline was predictable and similar to the earlier work of the author: *Greeting with Opening Music, Movement to Music, Singing, Instrument Playing, Closing Music* [11, p. 65].

After 2 years, the MT group grew to the point that it no longer was comfortable in the university session space, creating potential liability issues. The initial caregiver who advocated to begin the group suggested an expansion of the partnership to include the city. After several discussions and meetings with city government officials and the program director at the adult recreational center, it was agreed that the group would begin meeting in a room with a piano at the adult recreation center. The new space was a part of a large town center complex that included a library, history museum, arts center, and eventually became the home for a weekly memory café. Upon moving to the town center location, the program grew rapidly. Assisted living and memory care communities brought groups of residents for the monthly music therapy sessions. This was not in accordance with the original mission of the program designers, and it this larger group grew to between thirty and sixty people attending, and individual needs were not being met. The group rules were re-instated, and those who were already living in an assisted living residence were referred to qualified MT's to deliver services to their residential program. The majority of the remaining “care partner teams” residing at home requested to increase offerings to weekly sessions and the schedule was changed to meet this need and to coordinate with the academic year so that more student therapists could participate in the program as a practicum experience.

A resource-oriented approach evolved as the underlying theoretical framework, with relationship between care partners, group members, and facilitators at the forefront. The strengths of the individuals were emphasized, accentuating positive experiences, as well as providing a venue for expressing difficult emotions. Each individual's relationship with music was a primary consideration as well, and the preferred, meaningful music of each “care partner team” was highlighted within the sessions. Resource-oriented music therapy (29) involves the “nurturing of strengths, resources and potentials;” involves “equal collaboration rather than intervention;” views the individual “within their context;” and sees “music as a health resource” (p. 561).

### Session format

#### Opening music and greeting

An opening song was used to greet members, supporting relationship development and facilitating memory through rehearsal of names. “Oh, what a Beautiful Morning” became the most preferred greeting song, and after the original song lyrics were sung, participant's names were sung, along with providing nametags. This repeated opening helped each person feel welcome, and their identity was reinforced. Although the practice of singing names may be perceived as immature, it is important to note that the “care partner teams” were emphatic that their names be sung within the opening song each week. It was very helpful for participants with memory loss and new members of the group, and it became an effective way to introduce students, volunteers, and guests, while providing a unifying ritual to indicate the beginning of the session following individual greetings from group facilitators. “Current events” of participants were elicited, bringing support through shared experiences. One “care partner team” often arrived late due to distance and difficulty in getting the PwD ready. Sharing this experience of typical morning stressors opened the door for others to share difficulties, allowing peers to listen and empathize. This act of sharing and listening helped reduce the stress of the participants, building camaraderie and rapport.

### Physical movement

Gestures, dance, and gentle movement were always used, adapted from yoga, neurologic music therapy (30), and from dance styles. As much as possible, this part of the session included orientation and exploration of the environment. This part of the session might begin with hand shaking among participants, deep breathing exercises, and making observations on the environment. This mindful observation allowed for awareness of those present, as well as participants who weren't in attendance, which would often lead to conversation on recent events and updates on group members' activities outside of the MT group. These observations and conversations strengthened relationships and allowed opportunities for leadership among the peer group. Caregivers took ownership of the group, emailing and phoning to check in on care partners who were not in attendance.

### Singing

Familiar, novel, and newly composed songs provided therapeutic outcomes. Songs that were well known offered opportunities to maintain vocal skill and active participation, stimulating language and memories. Novel songs allowed for new learning, and provided a way for care partners to share their identity as a couple. This personal identity is exemplified in songs specific to the region of the participants' origin, which allowed the care partner's younger and healthier identity to be shared with the group. A state song, a school song, a song about a time of life, courtship or dance were re-created and performed. Lyric sheets were provided for the group members and the song list was periodically updated to include member favorites, with the assistance of caregivers. These songs specific to the couple became “theme songs” and care partners spontaneously held hands, sang along and expressed emotions as “their song” was sung for them. The songs became important in maintaining personal identity, with some participants requesting their favorite song each session, and peers remembering each other's songs and stories of importance. Spiritual music was regularly requested, especially “Amazing Grace” as well as patriotic music that was known by all. Songs were newly composed during the session to provide a way to help express thoughts and feelings, as well as to offer choices. There was often an improvised song as a way to include music that each participant could participate in to “amplify” their comments and contributions. For example, if a participant mentioned that they had been ill during the week, an improvised chant with a known or newly created melody, would be created around a simple phrase, such as “we're glad you're feeling better.” If there was a loss of a friend or family member, this time in the session would be acknowledge the loss, and the newly created music used for comfort. Birthdays and anniversaries were celebrated, and there was a flow between improvising music to accompany and deepen the meaning behind the words, and a return to words for further exploration. This was also a way to provide a unifying theme for the remainder of the session, and a touch point to return to if there was confusion.

### Instrumental play

Each week, participants were provided with opportunities to manipulate and choose instruments. Instruments that were most frequently available were hand percussion, such as drums, rainsticks, tambourines, and maracas. Special instruments were introduced, such as tone chimes, thunder tubes, glockenspiels, as well as opportunities to listen to instruments played by others. Student therapists brought a variety of orchestral, folk, and unique ethnic instruments from many cultures for listening and discussing. Group members played piano to accompany singing, and guitar was used each week by the MT facilitators. The instruments were used for improvisation, rhythm, sound effects, and sensory awareness, as well as cultural experiences and a way to make new associations and connections as an intergenerational experience.

### Creative space

Since the space was housed within an active adult recreational center, there were many programs taking place before and after the MT group, as well as open access to a gym and outdoor patio. There were arts groups where curious individuals were invited to join in with singing, and a regular volunteer vocalist came in to perform for and with the MT group. The creative space provided close contact with community neighbors, musicians, and creative artists, as well as easy proximity to arts events and fundraising for community outreach taking place within the same complex. The support group had developed its own identity as a welcoming place, connected to community.

### Verbal conversation

To allow for the most participation, conversation was interspersed throughout the session, and was most fruitful toward the end of the session, when all parties had experienced active music making and had heard their own voice, spoken or sung, and had become engaged in the active process of participation. For those who were unable to speak clearly or coherently, the care partner or another participant provided their own understanding of what was being communicated, and then the facilitators would reflect this back to the group in an animated way with increased volume, so that others could hear and respond.

### Closing

Singing a familiar song with a moment to reflect on group achievements provided a way to prepare for departure. Songs that were most used were: Happy Trails, Goodnight Sweetheart, So Long Farewell, Bye Bye Blackbird, Goodnight Ladies, God Bless America, and America the Beautiful. It was regularly observed that by the end of the session, the care teams had more relaxed expressions and posture, and informal socialization continued among care partner teams in the session room and lobby.

### Outcomes-empowerment and self-initiation

Through observations and self-reporting by care partners, it became clear that many of the initially identified needs were being met by the members of the group. In the third summer of the MT program, the group was scheduled to be on break for 3 summer months. Members of the group had become close, and one of the couples who had been participating regularly and lived near the community center offered to continue meeting throughout the summer at their home. This was seen as especially important occurrence, and as it indicated that the group had bonded and become independent. It was not a replacement of the MT program, but an opportunity to come together for fun and companionship, and share music amongst themselves in the absence of professional facilitators.

The MT group underwent phases of membership, and there was a full spectrum of community dwellers who attended the group regularly. All the members were retired, and some had worked in community service and as educators, so felt comfortable as group leaders. Many had traveled and raised families, and although the town center was easily accessible by public transportation, the care partners were all able to drive to the sessions. It was not known if there were members of the group who were experiencing poverty or financial hardship, as demographic information was not requested.

There were opportunities for leadership for care partners and PwD. One care partner was an avid researcher of various techniques on movement for health, particularly for Parkinson's movement disorders to help her husband, and she facilitated movement for the group. Another PwD was a European immigrant with English as her second language. She graciously taught the group greetings and simple phrases in her language of origin, to which the group created tunes and sang. Another PwD was a retired dentist, and eagerly shared stories of his family life and work, telling jokes and providing a jovial attitude. Another care partner was an organizer and communicator, gathering contact information and sharing it with the network. She helped survey for group interests, and shared messages between group members as needed. The AD research and treatment center provided printed copies of favorite large print song lyrics, organized by table of contents, which were kept in the community center closet. The song books were maintained by participants, volunteers and students, and allowed for the group repertoire to be remembered and shared, added to as new members joined. All of these contributions maintain a resource-oriented approach to leadership, which empowers the members to have a voice in the direction of the program.

One of the caregivers provided her perspective, included here as a testimony:

*Our music therapy class has had a powerful impact on our lives. Our group consists of people with Alzheimer's Disease, Parkinson's or other neurological diseases, and their caregivers. Most caregivers are spouses, but some are adult children*.

We come together weekly from all over the county, some traveling an hour to get there. Yet we all make the effort every week because we have become close friends, and we love singing together and sharing our memories and feelings with one another…

*(We) sometimes get… up dancing to music (we) overcome our inhibitions and just move with joy to the rhythm of the music. We often play rhythm instruments as we sing our favorite old songs to the accompaniment of her guitar. Our loved ones respond…some don't normally talk, but she knows how to engage them individually, which is amazing to watch*.

*Several other caregivers and I have either lost our loved ones, or placed them in memory care facilities, yet we still attend music class because it is healing to our souls. It's a very important support group in our lives. We're so grateful for a safe a healing place for us and our loved ones*.

The MT group proved to be clearly sustainable, as it continues with new facilitators and new members in its seventh year. There have been two additional groups modeled after this program in new locations in 2016, at community member requests. A supervising MT has been able to develop these groups with the support of community partners, in donated or low-fee spaces, at the initiative of caregivers who advocate for creative services for their loved one with dementia and for their own support and the health and wellbeing of their loved one with dementia.

## Recommendations

From 2011 to 2018 there have been several transitions of participants and facilitators of this MT group. As participants became unable to attend due to health, moving to a residence or treatment program, or death, there was a consistent overlap of new participants looking for support and encouragement within the context of a community MT group. Caregivers came to visit and kept in touch with each other through phone calls and gatherings.

It is recommended that stakeholders and care providers learn more about the demographics of the community, and compare the MT support group members with overall demographics to recognize and reach members of the community who are not being served. It is likely that members of the community for whom English is a second language are not receiving information about the program. A possible partner for learning more about disadvantaged PwD may be *Meals on Wheels* or similar agencies that focus on low income elders.

It is recommended that the program be piloted as a research study to examine more thoroughly the outcomes for PwD and for caregivers. Clair (31) reports on the need for rigorous research that tests protocols in music therapy, including randomized controlled trials or other research methods with scholarly rigor. To provide a greater understanding of the effects of this type of group, researching the impact on caregiver burden, reduction in stress, or improvement in PwD symptoms by administering reliable and valid rating scales and data collection is recommended. Research would help shape the offerings for care partner teams and, if stronger outcome-based evidence is achieved, the program could become more accessible to those who need it. If evidence supports the need for more support for the caregiver, a separate “caregiver only” music therapy group could be offered to more specifically assist and provide respite to the caregiver while the PwD is enjoying a separate experience focused on their needs in the moment. Additionally, a focus on caregiver grief following death of their loved one could be the beginning of a new MT support group. Formal and frequent requests for input from caregivers could help provide valuable information on ways to improve offerings, rather than the less formal communication that was the norm in this community group. Screenings for depression and counseling service referrals could also be introduced, in an effort to detect more serious caregiver issues.

Finally, this type of support group, embedded in the community, offers ways to remain actively supportive of PwD and their caregivers as they need more assistance. The original member of the group, “Jackie,” was followed by a professional and student therapist, and provided individual MT when she was no longer able to benefit from the group. It is hoped that with increased collaboration between local government, creative arts, caregivers, and professionals in healthcare, that those affected by Alzheimer's and related dementias will have greater accessibility to music therapy and improved continuity of care as the disease progresses.

## Author contributions

The author confirms being the sole contributor of this work and has approved it for publication.

### Conflict of interest statement

The author declares that the research was conducted in the absence of any commercial or financial relationships that could be construed as a potential conflict of interest. The reviewer KSW and handling Editor declared their shared affiliation.
